# Characterization of a Novel Polysaccharide Lyase Family 5 Alginate Lyase with PolyM Substrate Specificity

**DOI:** 10.3390/foods11213527

**Published:** 2022-11-06

**Authors:** Licheng Zhou, Qing Meng, Ran Zhang, Bo Jiang, Xiaoyong Liu, Jingjing Chen, Tao Zhang

**Affiliations:** 1 State Key Laboratory of Food Science and Technology, Jiangnan University, Wuxi 214122, China; 2 International Joint Laboratory on Food Safety, Jiangnan University, Wuxi 214122, China; 3 Shandong Haizhibao Ocean Technology Co., Ltd., Weihai 264333, China

**Keywords:** alginate lyase, PL 5, catalytic residues, site-directed mutation, oligosaccharides, endolytic

## Abstract

Alginate lyases (ALyases) have been widely applied in enzymatically degrading alginate for the preparation of alginate oligosaccharides (AOS), which possess a range of excellent physiological benefits including immunoregulatory, antivirus, and antidiabetic properties. Among the characterized ALyases, the number of ALyases with strict substrate specificity which possess potential in directed preparation of AOS is quite small. ALyases of polysaccharides lyase (PL) 5 family have been reported to perform poly-*β*-D-mannuronic acid (Poly-M) substrate specificity. However, there have been fewer studies with a comprehensive characterization and comparison of PL 5 family ALyases. In this study, a putative PL 5 family ALyase PMD was cloned from *Pseudomonas mendocina* and expressed in *Escherichia coli*. The novel ALyase presented maximum activity at 30 °C and pH 7.0. PMD displayed pH stability properties under the range of pH 5 to pH 9, which retained more than 80% relative activity, even when incubated for 48 h. Product analysis indicated that PMD might be an endolytic ALyase with strict Poly M substrate specificity and yield disaccharide and trisaccharide as main products. In addition, residues K58, R66, Y248, and R344 were proposed to be the potential key residues for catalysis via site-directed mutation. Detailed characterization of PMD and comprehensive comparisons could supply some different information about properties of PL 5 ALyases which might be helpful for its application in the directed production of AOS.

## 1. Introduction

As a renewable resource, alginate is a natural anionic polysaccharide occurring in the cell walls and intracellular material of brown algae. Meanwhile, it is an important source of fixed carbon in marine ecosystems [1] and has been widely applied in the food industry, serving as a viscosifier, stabilizer, and gelling agent. In addition, alginate oligosaccharides (AOS), as the degraded products of alginate with high solubility and low viscosity, have been reported to possess a wide range of outstanding biological activities which expand the application fields of alginate [2]. Therefore, alginate lyases (ALyases) catalyzing the degradation of alginate via a *β*-elimination mechanism have been gaining increasing attention nowadays [3]. There have been numerous reports about exploring the application of enzymes derived from microbial sources. For example, a type of L-asparaginase (HR03Asnase) has been cloned from marine *Pseudomonas aeruginosa* and its potential applications in the food industry and the treatment of cancer have been evaluated [4]. ALyases are widely distributed in nature and are typically obtained from various bacteria and fungi, marine algae, and mollusks [5]. According to amino acid sequence analysis, ALyases fall into 14 PL families (PL 5, -6, -7, -8, -14, -15, -17, -18, -31, -32, -34, -36, -39, and -41) in the Carbohydrate-Active enzymes (CAZY) database [6]. Based on the substrate specificity, ALyases have been classified into three categories: poly-*β*-D-mannuronic acid (PM) specific lyases (EC 4.2.2.3) preferring degrading M block in alginate, poly-*α*-L-guluronic acid (PG) specific lyases (EC 4.2.2.11) preferring degrading G block in alginate, and bifunctional lyases (EC 4.2.2.-) degrading both M and G block in alginate without evident preference [7]. Regretfully, this definition is still not comprehensive. For example, polyMG-specific lyases are not taken into consideration. In addition, based on their modes of action, ALyases have been categorized into two classes: endo-alginate lyases and exo-alginate lyases. Endolytic ALyases cleave glycosidic bonds from the inside chain of alginate polymers and release unsaturated alginate oligosaccharides with different degrees of polymerizations (DPs) as main products, while exolytic ALyases can only degrade alginate molecules into monomers or dimers from the ends of the chains [8,9,10].

As the main products of alginate degradation, AOS have been reported to possess many outstanding physiological functions, such as immunomodulatory activity, antimicrobial activity, antioxidant activity, antitumor activity, prebiotic activity, antihypertensive activity, and antidiabetic activity [11]. Based on the monomer sequences, AOS have been divided into three types: oligomannuronate (MAOS), oligoguluronate (GAOS), and heterozygous AOS (HAOS). Further, according to the reported literature, the biological activities of AOS might be closely related to their composition and structure. For example, GAOS has been reported to specifically bind to the bacterial surface and regulate the surface charge. As a result, microbial aggregation was induced to occur and bacterial motility was inhibited [12]. It was unlikely that MAOS (100% M; molecular weight: 3000–3500 Da) showed less biofilm regulatory activity than GAOS [13]. However, studies focusing on the biological functions of enzymatic producing AOS seldom shed a precise light on the relationship between structural composition and biological functions. This might be attribute to the challenge on directed enzymatic production of AOS with specific composition and structure. Recently, there have been several reports on the production of AOS with ALyases. Regrettably, their structure and composition were always random, which might be linked to the source of substrate, action mode and substrate specificity of ALyases applied, reaction conditions, and so on [14]. Among them, substrate specificity of ALyases might be one of the important factors. Therefore, compared with bifunctional ALyases, ALyases with strict substrate specificity might be a useful tool to be helpful in the directed production of AOS. There have been reports that PL 5 ALyases are a member of PM specific lyases with strict Poly M specificity (activity can not be detected with poly-*α*-L-guluronic acid as substrate) which are rare in characterized ALyases. However, when it comes to the literature, there have been fewer studies with comprehensive characterization and comparison of PL 5 family ALyases. As shown in Table 1, a total of 12 characterized PL 5 family ALyases and their enzymatic properties have been listed here. Among them, 58% were identified prior to the 21st century and information about their enzymatic properties was quite short, such as algA (GenBank No. BAA33966.1) from *Deleya* marina and algL (GenBank No. AAA71990.1) from *Pseudomonas aeruginosa* [15,16]. Furthermore, to date, there have only been three PL 5 ALyases whose crystal structures have been solved and the critical catalysis residues (even the potential ones) of PL 5 ALyases are seldom confirmed via experiments that would provide more information to study the interaction between substrate and catalytic pocket. Hence, a detailed characterization of a new PL 5 family ALyase and comprehensive comparisons might be of some interest and supply some different information about the properties of PL 5 ALyases which might be a potential useful tool for the directed production of AOS.

In this study, a putative PL 5 family ALyase code gene PMD (sourced from *Pseudomonas mendocina*, GenBank No. WP_047587958.1) was obtained from GenBank Database and expressed in *Escherichia coli* (BL21). The enzymatic properties of PMD were identified and comparisons were performed. PMD was characterized as an endolytic ALyase with strict Ploy M specificity and performed well in pH adaptation properties. Product analysis indicated that PMD might yield disaccharide and trisaccharide as main products. With the assistance of molecular docking (Autodock version 4.2, San Diego, California, CA, U.S.A), the potential critical catalytic residues were also identified via site-directed mutation.

## 2. Materials and Methods

### 2.1. Plasmids and Materials

The coding gene of putative ALyase PMD (GenBank No. WP_047587958.1) was generated from GenBank Database. The sequence of gene with an in-frame C-terminus fusion 6×histidine-tag was synthesized by Sangon Biotech (Shanghai, China) and inserted into the pET28a (+) vector between the *Nde* Ⅰ and *Xho* Ⅰ site. *E. coli* DH5*α* and *E. coli* BL21 (DE3) were used for gene cloning and expression, respectively. PrimeSTAR MIX DNA polymerase, Taq DNA polymerase, Dpn Ⅰ was purchased from TaKaRa (Dalian, China). DNA products were purified via DNA purification kit purchased from Novagen (Darmstadt, Germany). Sodium alginate (M/G ratio 1.87) was purchased from Sangon Biotech (Shanghai, China). Polymannuronate (PM, purity: ~99%), polyguluronate (PG, purity: ~99%), and glucuronic acid standards (monomer, dimer, and trimer) were purchased from Qingdao BZ Oligo Biotech Co., Ltd. (Qingdao, China).

### 2.2. Sequence Analysis and Homology Modeling

The ProtpParam tool provided by ExPaSy server of the Swiss Institute of Bioinformatics (https://web.expasy.org/protparam/ (accessed on 14 August 2021).) was applied to estimated molecular weight and theoretical pI of PMD. Phylogenetic analysis of PMD was carried out via MEGA (version 5.0, Mega Limited, Auckland, New Zealand) [27]. The amino acid sequence of PMD was subjected to BLAST analysis (https://blast.ncbi.nlm.nih.gov/Blast.cgi (accessed on 14 August 2021).) and the seven reported PL 5 family alginate lyase sequences (sequence identity more than 50%) were aligned using ClustalW (http://www.ebi.ac.uk/clustalw/ (accessed on 14 August 2021).) [28].

The homology models of PMD were constructed via SWISS-MODEL (https://swissmodel.expasy.org/SWISS-MODEL.html (accessed on 15 August 2021).) using alginate lyase AlgL (sourced from *Pseudomonas aeruginosa* PAO1, PL 5, PDB: 4OZV) as the template [29,30].

### 2.3. Recombination Strains and Site-Directed Mutations

The whole plasmid polymerase chain reaction (PCR) method was used to perform site-directed mutation which contained 50 ng template in total (pET-28a(+)-PMD), 0.1 μM of each primer (example: forward primer 5′-TACTAGCgctTACGAGGGGTCTAACTCGGCTC-3′ and reverse primer 5′-CCTCGTAagcGCTAGTAAACTGCAAATCGCCA-3′used for K58A site-directed mutation), 10 μL PrimeSTAR MIX and ddH_2_O up to 20 μL. PCR cycle conditions were: initial denaturation for 5 min at 95 °C, followed by 26 cycles of 15 s at 98 °C, 30 s at 52–65 °C, 190 s extension at 72 °C. Then, 1 μL Dpn Ⅰ enzyme and 2 μL buffer were added into the mixture to digest parental methylated and hemi-methylated DNA at 37 °C for 2 h. The purified products were finally transformed into *E. coli* DH5*α* competent cells and incubated under the condition of 37 °C and 200 rpm for 1 h. The incubated mixture was coated on an Luria-Bertani (LB) solid plate which was cultivated at 37 °C. After overnight cultivation, the recombinant strains were verified by sequencing. Primers used in this study are listed in Table 2.

### 2.4. Expression and Purification of PMD and Mutants

Tubes with 5 mL LB medium were used to inoculated single colonies and then cultured under the condition of 37 °C and 200 rpm for about 12 h. Then, each tube was inoculated into 200 mL terrific broth (TB) medium (1 mL) and cultured at 37 °C with shaking at 200 rpm until the OD_600_ reached 0.6. Isopropyl-*β*-D-thiogalactopyranoside (IPTG) was added (1 mM final concentration) and cultured at 20 °C by shaking at 200 rpm to induce the expression of alginate lyase. After 24 h of inducing, the culture medium was then centrifuged under the condition of 6000 rpm for 15 min. The supernatant was decanted while the cells were collected and washed with deionized water. Next, the collected cells were resuspended in 15 mL lysis buffer (20 mM Tris-HCl, 100 mM NaCl, pH 8.0) and lysed via an ultrasonic disruptor for 20 min [31]. Then, the mixture was centrifuged again under the condition described above. Ultimately, the supernatant containing alginate lyase with histidine tag was purified via Ni-NTA chromatography. The purity of the proteins was tested via 12.5% sodium dodecyl sulfate-polyacrylamide gel electrophoresis (SDS-PAGE) [32]. Finally, the purified enzyme solution (about 2 mL) was dialyzed against 10 mM Tris-HCl buffer (pH 7.0, 1 L) for 24 h and the dialysis buffer was changed every 6 h for four times. Ethylene Diamine Tetraacetic Acid (EDTA, 5 mM final concentration) was added to the dialysis buffer at the first time to isolate metal ions in the purified enzyme solution. EDTA-pretreated purified enzymes were stored in an ultra-low temperature refrigerator at –80 °C.

### 2.5. Enzymatic Activity Assay of PMD and Mutants

The standard reaction mixture (1 mL) consisted of 0.5% (*w/v*) substrate (sodium alginate, PM, or PG), 500 mM sodium chloride, 10 mM phosphate buffer (pH 7.0), and 100 μL crude enzyme solution, or 4 μg purified enzyme protein. The 3,5-dinitrosalicylic acid (DNS) method with D-glucose as standard was applied to assay alginate lyase activity [33]. The reaction was terminated by boiling for 10 min. Afterward, 150 μL reaction mixture together with 500 μL DNS were boiled for 10 min. Finally, 500 μL H_2_O was added and measured at the absorption of 540 nm. One unit (U) of alginate lyase activity was defined as the amount of enzyme required to produce 1 μmol of unsaturated product per min at 30 °C and pH 7.0.

### 2.6. Biochemical Characterization of PMD

To determine the optimal reaction temperature, the activity of PMD was evaluated at 4–50 °C (pH 7.0). The thermal stability was determined via measuring the residual activities of the enzyme incubated at 30 °C, 35 °C, 40 °C, and 45 °C separately for different times. The optimal pH was determined at 30 °C with different 50 mM buffer systems: sodium citrate buffer (5.0–6.0), phosphate buffer (6.0–8.0), Tris-HCl buffer (7.5–8.5), glycine-NaOH buffer (8.5–9.5) [34]. After 12~48 h incubation in different pH buffers, residual enzyme activity was determined to evaluate pH stability of PMD. The effect of different concentrations of NaCl on the activity of purified enzyme was determined via evaluating residual activities at the optimum reaction condition with different NaCl concentrations (from 0.05 to 2.0 M). Parallel reactions without NaCl were regarded as controls. To measure the effects of other metal ions, the EDTA-pretreated purified enzymes were incubated with 1 mM of various metal ions (Fe^3+^, Fe^2+^, Co^2+^, Ni^2+^, Cu^2+^, Zn^2+^, Ca^2+^, Mg^2+^, Mn^2+^, Na^+^, and K^+^) at 4 °C overnight. The activity of the control was defined as a relative activity of 100% [35].

### 2.7. Substrate Specificity and Kinetic Parameters

To determine the substrate specificity of PMD, the enzyme activity was measured with three kinds of polymer solutions (sodium alginate, PM, PG) whose final concentration was 0.5% (*w/w*).

The kinetic parameters of PMD was assayed under the optimum reaction conditions and the concentration of substrate range from 1 mg mL^−1^ to 10 mg mL^−1^. Kinetic data were determined via the Hanes–Woolf plot equation [36].

### 2.8. Product Analysis

#### 2.8.1. Liquid Chromatography-Mass Spectrometry (LC-MS) Analysis

The reaction mixture including 20 μg purified enzyme and 0.1% (*w/v*) sodium alginate was maintained under the optimum condition for 0~24 h and terminated via a 10 min boiling water bath. The supernatants were collected after centrifugation at 12,000 rpm for 10 min. The enzymatic hydrolysis process of PMD was monitored by liquid chromatography–mass spectrometry (LC-MS) with an ultra-performance liquid chromatograph, a photo-diode array detector and a BEH C18 column (WATERS, Milford, MA, USA). The temperature of the column was set at 45 °C. Pure acetonitrile and formic acid with the concentration of 0.1% (*w/w*) was used as Phase A and B, separately. The gradient elution process was as follows: 100% B for 40 min, 30% A and 70% B for 45 min, 80% A and 20% B for 50 min, 100% A for 55 min, and 100% B to the end. The flow rate was 0.3 mL min^−1^ all the time. When it came to second stage, the electrospray ionization source was used as ion source during MS analysis and other conditions were set as capillary voltage of 3.5 kV, cone voltage of 30 V, source block temperature 100 °C, desolvation temperature 400 °C, and a mass scan from 20 to 2000 *m/z* [37].

#### 2.8.2. Thin-Layer Chromatography (TLC) Analysis

The reaction mixtures including 10 μg purified enzyme and 0.25% (*w/w*) different types of substrate (PM, PG, and sodium alginate) were maintained under the optimum condition for 6 h and terminated via a 10 min boiling water bath. The supernatants were collected after centrifugation at 12,000 rpm for 10 min. The hydrolysis products were analyzed by thin-layer chromatography (TLC). The 1 μL supernatants mentioned above were developed with 1-butanol: acetic acid: ddH_2_O (3:2:3, *v/v*), and visualized by heating at 120 °C after spraying with sulfuric acid: ethanol (1:9, *v/v*). The mixture without adding enzyme was regarded as the control.

### 2.9. Molecular Docking

The rigid complex of PMD and tetrasaccharide was constructed via AutoDock 4.2. The structure of a mannuronic acid tetramer was obtained from PDB (https://www.rcsb.org/ (accessed on 20 August 2021).) and the energy of it was minimized via Chem3D (version 17.0, PerkinElmer, Waltham, MA, USA). The PMD molecule was restrained within a grid box (70 × 70 × 60 points in each dimension). The docking searches were performed via the Lamarckian genetic algorithm with the default settings for all options [38]. It was docked for one hundred times and a total of 1000 complexes ranked by binding energy were finally generated.

### 2.10. Circular Dichroism (CD) Spectra

To determine the secondary structure of PMD, a CD spectra (Applied Photophysics Ltd., London, UK) was performed. The data were collected every 1 s at an interval from 260–190 nm and three repeats were averaged. The concentration of PMD was diluted to about 0.1 mg mL^−1^ using the final dialysis buffer which also was used as the control. The secondary structure of PMD was calculated via CDNN software (http://thelab.photophysics.com/circular-dichroism/protein-secondary-structure-analysitools-cdnn/ (accessed on 20 August 2021)).

## 3. Results and Discussion

### 3.1. Cloning, Expression and Purification of Mutants

The putative alginate lyase PMD encoding gene (GenBank No. WP_047587958.1) was generated from NCBI GenBank, which was a fragment of the whole-genome shotgun sequence of *Pseudomonas mendocina* ZWU0006 (NCBI Reference Sequence: NZ_JTLK01000209.1). The PMD encoding gene together with an added 6×histidine-tag located at C-terminus was synthesized and inserted into pET-28a (+) vector. Then, it was transformed into *E. coli* BL21 (DE3) and induced with IPTG (final concentration: 1 mM). As shown in Table 3, the purification procedure was summarized here. The activity of the crude enzyme was 8.16 U mL^−1^ and 11.74-fold purification was achieved with 88.14% yield through one step of Ni-NTA chromatography. Finally, the activity of the purified enzyme was determined as 44.97 U mg^−1^. In addition, the purified enzyme showed an apparent band on the SDS-PAGE gel (Figure S1), indicating the purity of the enzyme was adequate to characterize the enzymatic properties [25].

### 3.2. Sequence Analysis, Sequence Alignment and Homology Modeling

**Figure 1:**
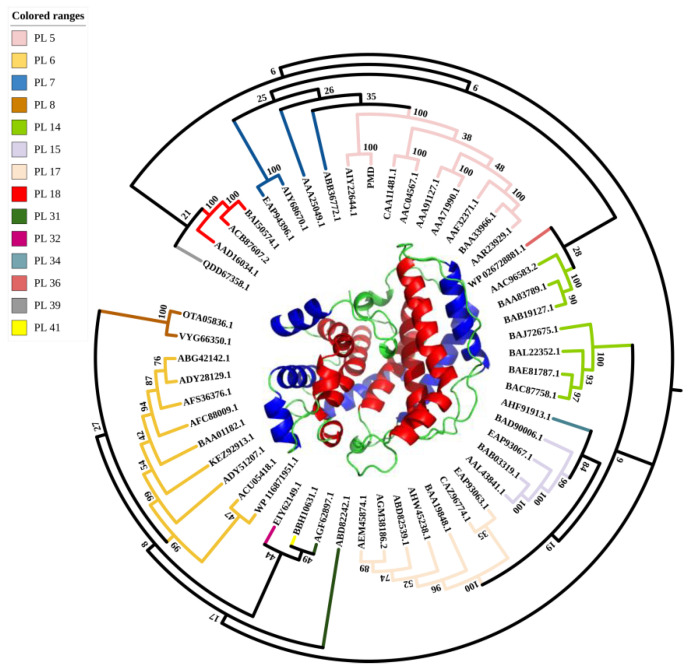
Phylogenetic tree analysis of reported alginate lyases from various microorganisms. The numbers at the branching points were the bootstrap values. The color of the branches indicated the classification of alginate lyases. The tree was created by the neighbor-joining method with MEGA-5 software and further modified via the ITOL web service. The structure of the novel alginate lyase (PMD) was prepared using the SWISS-MODEL web server and colored in the rule (inner helix: red, outer helix: blue, and others: green).

**Figure 2:**
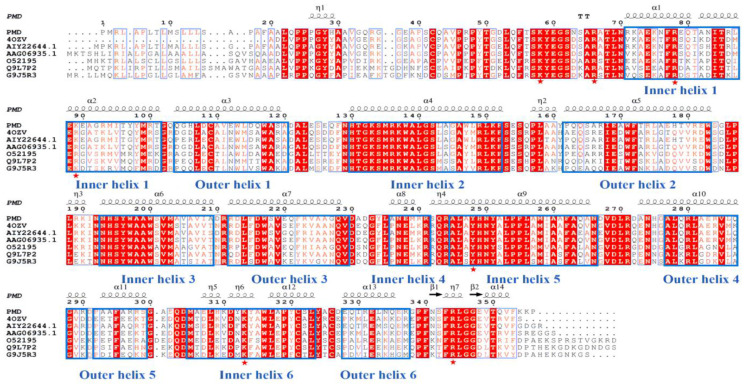
Multiple sequence alignment with structure prediction of the novel alginate lyase (PMD) and six reported polysaccharide lyase (PL) 5 family alginate lyases. The conserved amino acid residues are shown in red. The structure regions were labeled according to (*α*/*α*)6-half-barrel rule (inner and outer layer). The mutated residues K58A, R66A, R78A, K89A, Y248A, K313A, and R344A are labeled with red pentastars.

The open reading frame (ORF) of PMD consisted of 1074 bp encoding protein of 357 amino acids. In addition, the G+C content of PMD was 54% overall. The amino acid sequence was submitted to the ProtpParam tool provided by ExPaSy server of the Swiss Institute of Bioinformatics and its theoretical molecular weight and pI were calculated to be 38.02 kDa (consistent with Figure S1) and 8.83, respectively. As shown in Figure 1, sequences of alginate lyases from 14 PL families were used to construct the phylogenetic analysis and PMD falls into a PL 5 family. Among the PL 5 family, AlgA (GenBank No. AIY22644.1, PL 5, generated from *Pseudomonas* sp. E03) has the nearest relationship with PMD while Alyase like algA (GenBank No. BAA33966.1, PL5, generated from *Deleya* marina) has the farthest evolution relationship with PMD. In addition, the amino acid sequence alignment was carried out via ClustalW(http://www.ebi.ac.uk/clustalw/ (accessed on 14 August 2021).), as described above (Figure 2) [39]. The putative alginate lyase PMD shared 86.6%, 61.8%, 60.9%, 56.7%, 54.2%, and 51.3% amino acid sequence similarity with AIY22644.1 [25], 4OZV, AAG06935.1 [17], O52195 (GenBank No. AAC04567.1) [18], Q9L7P2 (GenBank No. AAF32371.1) [21], and G9J5R3 (GenBank No. AEW23144.1) [23], respectively. Sequence analysis (Figure 2) together with homology modeling (Figure 1) via Swiss-model using the crystal structure of AlgL (PDB: 4OZV) as the template showed that, the same as AlgL, PMD mainly carried *α*-helix in its secondary structure which was confirmed by CD analysis result (Figure S2). Helix occupied about 85.3% of the total secondary structure of PMD (Table S1). PMD possessed the central (*α*/*α*)6-half-barrel catalytic domain which consisted of two layers of *α*-helices. The inner-layer (red) and outer-layer (blue) *α*-helices were labeled in Figure 1. Furthermore, when compared to multi-domain PL 15 alginate lyase such as Atu3025, the complete conformation of PMD was much tighter. As a result, PMD seemed similar to a half barrel where the catalytic pocket lay on it. As displayed in Figure 2, the red colored residues were the most conserved ones, which might play important roles in enzyme catalysis. For example, the conserved residue H257 of *Vibro cholerae* flavin transferase ApbE was the critical residue for substrate binding and catalysis [40].

### 3.3. Enzymological Characterization of PMD

#### 3.3.1. Effects of Temperature and pH

**Figure 3:**
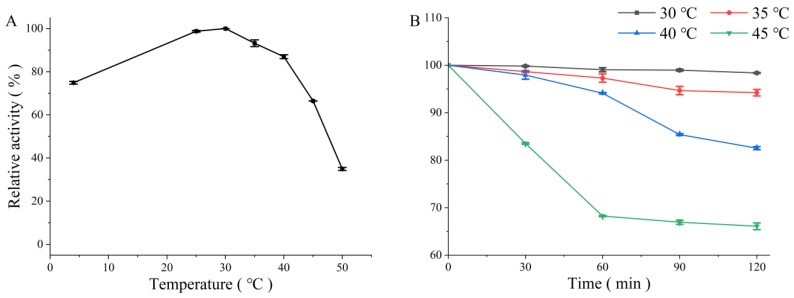
Effects of temperature on the enzymatic activity of the novel alginate lyase (PMD). (**A**) Activities of PMD under different temperatures (ranging from 4 °C to 50 °C); (**B**) thermostability of PMD.

The optimum temperature of PMD was shown at 30 °C and it presented over 70% of the maximum activity at temperatures ranging from 4 °C to 40 °C (Figure 3A) reflecting its adaptability in this temperature region. The thermal stability of PMD was determined via measuring the residual activities of the enzyme incubated at 30 °C, 35 °C, 40 °C, and 45 °C. As shown in Figure 3B, PMD possessed more than 90% relative activity at 30 °C and 35 °C after incubation for 2 h. When it came to 40 °C and 45 °C, it still possessed more than 80% and 70% relative activity, separately. Compared with the other PL 5 family alginate lyases such as KS-408 alginate lyase obtained from *Pseudomonas* sp. strain KS-408 which almost totally lost activity after 30 min incubation at 40 °C [23], PMD performed greater thermostability at temperatures ranging from 4 °C to 40 °C. The enzymatic production of AOS is usually performed under the optimum conditions of applied enzyme. High temperature contributes to not only the increase in alginate solubility which helps to improve the product but also preventing microbial contamination. Hence, PMD with greater thermostability might be a more suitable candidate for thermal stability modification to finally generate a heat-resistant mutant which possesses potential application in directed AOS production among ALyases from the PL 5 family. 

**Figure 4:**
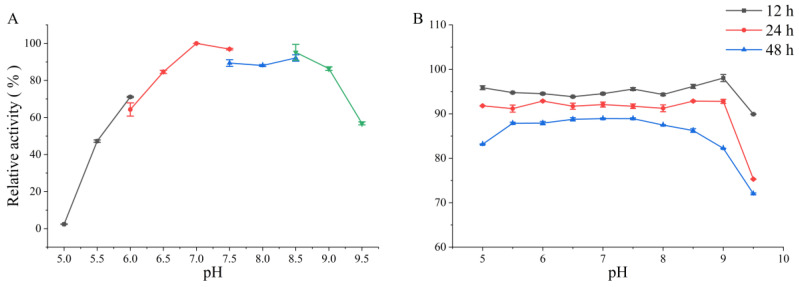
Effects of pH on the enzymatic activity of the novel alginate lyase (PMD). (**A**) Effect of pH on the enzymatic activity of PMD (grey: sodium citrate buffer, pH 5.0–6.0; red: phosphate buffer, pH 6.0–8.0; blue: Tris-HCl buffer, pH 7.5–8.5; green: glycine-NaOH buffer, pH 8.5–9.5); (**B**) pH stability of PMD.

The optimum pH of PMD was determined over the pH range of 5.0 to 9.5. As is shown in Figure 4A, the enzyme possessed an optimum pH of 7.0, and more than 60% of the highest enzyme activity was retained at a pH ranging from 6.0 to 9.0 while other PL 5 alginate lyases such as algL derived from *Pseudomonas* sp. QD03 only possessed more than 50% relative activity at a pH ranging from 7.0 to 8.0 [22]. This great pH-adaptation property might provide more leeway in the choice of pH application when it came to industrial produce. Meanwhile, PMD also showed pH stability at the pH ranging from 5.0 to 9.5, which showed more than 70% relative activity after 48 h incubation (Figure 4B).

#### 3.3.2. Effect of Sodium Chloride

**Figure 5:**
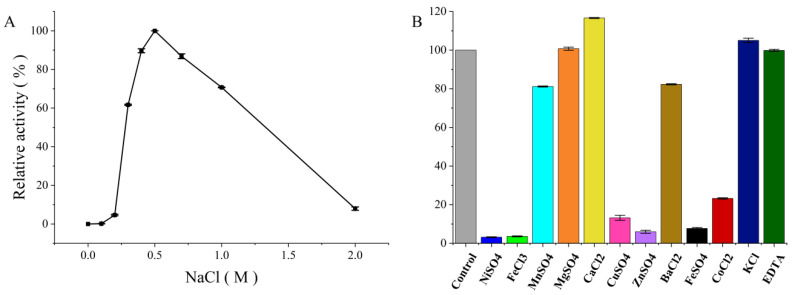
Effects of metal ions on the enzymatic activity of the novel alginate lyase (PMD). (**A**) Effect of NaCl on the enzymatic activity of PMD; (**B**) effects of metal ions on the activity of PMD.

Several alginate lyases, such as AlgL17 (theoretical pI: 5.72) and Aly08 (theoretical pI: 4.57), have reported that the enzymatic activity was related to the NaCl concentration in reaction mixture [41,42]. Similarly, as shown in Figure 5A, PMD still possessed more than 70% catalytic activity under 1 M NaCl and presented the max catalytic activity under 0.5 M NaCl. However, if no NaCl or other monovalent cations presented in the reaction system, the catalytic activity of PMD could not be detected which suggested that PMD was salt-activated alginate lyase. Interestingly, it was reported that Na^+^ only exhibited a slight activation effect on the activity of AlgA (GenBank No. AIY22644.1, PL5, generated from *Pseudomonas* sp. E03) and it still possessed detective activity without NaCl or other monovalent cations using a similar modified DNS method. In addition, the amino acid sequence of AlgA presented more than 86% sequence similarity with PMD [25]. Therefore, with the high sequence similarity and quite different properties toward sodium chloride, it might be a potential object to investigate the relationship between enzyme conformation and the effect of sodium chloride.

#### 3.3.3. Effect of Other Metal Ions

As displayed in Figure 5B, 11 types of metal ion and EDTA were added into reaction mixture at the concentration of 1 mM to investigate their influence on enzyme activity. Among them, the activity of PMD was slightly enhanced by Ca^2+^ and K^+^, while Mg^2+^ and EDTA seemed like special cases which almost showed no difference with the control. One possible reason for the enhancement of enzyme activity by metal ions is that metal ions might play the role of cofactors to participate in the enzymatic catalysis reaction or weaken the ionic interactions between alginate and enzyme [3]. For example, the substrate affinity of alginate lyase AlgAT0 was reported to be improved by tight binging to Ca^2+^ via one amino acid substitution [43]. In addition, the complexation constants of metal ions might be another important factor which might determine the formation of the intermediate complex formed by the enzyme, the substrate, and the metal ions. Ca^2+^ and Mg^2+^ are members of alkaline earth metal, and whose complexation constants are bottom-ranked among these divalent cations which might lead to the instability of intermediate complex. Based on the foregoing possible factors, the effects of Ca^2+^ and Mg^2+^ on PMD activity occurred. Mn^2+^ and Ba^2+^ displayed a negative influence on the activity of PMD with about a 20% decrease. Metal ions such as Ni^2+^, Cu^2+^, Zn^2+^, Fe^2+^, Co^2+^, and Fe^3+^ almost inactivated PMD with no more than 20% relative activity retaining. This might be due to the stable intermediate complex formed by the enzyme, the substrate, and the metal ions. In more detail, the metal ion might act as a noncompetitive inhibitor by noncompetitively binding to chemical groups to form a stable intermediate complex that cannot be decomposed and inhibits the activity of the enzyme [44]. The result of PMD was partially the same as characterized by PL 5 alginate lyases. Activities of AlgL from *Azotobacter vinelandii* and *Azotobacter chroococcum* were both slightly enhanced by metal ions such as Ca^2+^ and Mg^2+^, while activity of Smlt1473 discovered from *Stenotrophomonas maltophillia* was inhabited by all metal ions [18,20,24].

### 3.4. Kinetic Parameters and Substrate Specificity

To determine the substrate specificity of PMD, the enzyme activity was measured with three kinds of appropriate diluted polymer solutions (sodium alginate, PM, PG). As shown in Figure S3, PMD degraded both sodium alginate and PM. When compared together, the relative activity of PMD toward PM was only slightly higher than sodium alginate, which insists that PMD might also cleave glycosidic bonds between poly-*β*-D-mannuronic acids and poly-*α*-L-guluronic acid. However, when it came to PG, the activity could hardly be detected. Interestingly, this result was different from AlgA described above which possessed PM-dominating substrate specificity with only 14% relative activity towards sodium alginate [25]. This interesting phenomenon might provide an opportunity to figure out the relationship between substrate specificity and enzyme structure. Moreover, the strict substrate specificity of PMD would be helpful in polyM alginate oligosaccharides direction preparation.

The kinetic parameters of PMD with sodium alginate and PM as substrate were determined under the optimum condition via the Hanes–Woolf plot equation, and the results are presented in Table 4. The *K*m value of PMD using sodium alginate and PM as the substrate were 4.03 ± 0.68 and 1.32 ± 0.16 mg mL^−1^, respectively. The result suggested that the affinity toward the PM was higher than sodium alginate which might mean the binding rate of PM is better than sodium alginate. However, the values of *V*_max_ and *k*_cat_ were almost the same. One possible reason for this result might be that the higher substrate concentration of sodium alginate decreases the distance and increases the probability of collision between substrate and enzyme. Furthermore, in agreement with the *K*m value, when it came to the catalytic efficiency (*k*_cat_/*K*_m_), the value of PMD using sodium alginate as substrate was 57.57 mL mg^−1^ s^−1^, which was almost only one third of PM (169.70 mL mg^−1^ s^−1^). 

Given the factor that the activity of enzyme was not clearly characterized in most reports of PL 5 family alginate lyases and the methods of enzyme activity assay were different, it was difficult to make a comprehensive comparison within PL 5 family alginate lyases. However, with the few options, we were surprised to find that the activity of purified PMD (44.97 U mg^−1^) was almost 36.5-fold of purified AlgA whose activity was 222 EU mg^−1^ (EU: the amount of enzyme required to release 1 μg reducing sugar per min, 1 U = 180.16 EU). The *K*m value of AlgA was 28.5 mg mL^−1^ which was 21.6 times of PMD [25].

### 3.5. Product Analysis 

**Figure 6:**
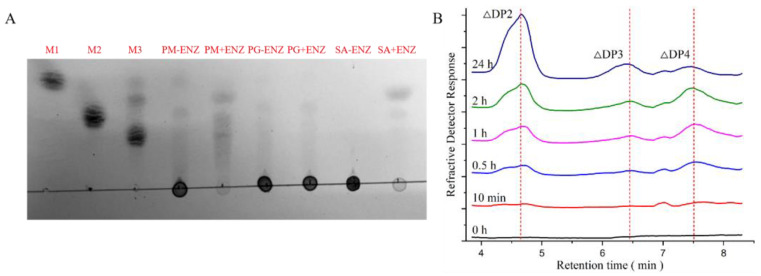
Product analysis of the novel alginate lyase (PMD). (**A**) Thin-layer chromatography (TLC) analysis of the final degraded products (6 h) by PMD (M1: mannuronic acid monomer, M2: mannuronic acid dimer, mannuronic acid trimer, +ENZ: with PMD, -ENZ: without PMD, SA: sodium alginate); (**B**) liquid chromatography (LC) analysis of the degraded products (sodium alginate used as substrate) by PMD ranging from 0 h to 24 h (peak ∆DP2: disaccharides, peak ∆DP3: trisaccharides, peak ∆DP4: tetrasaccharides).

**Figure 7:**
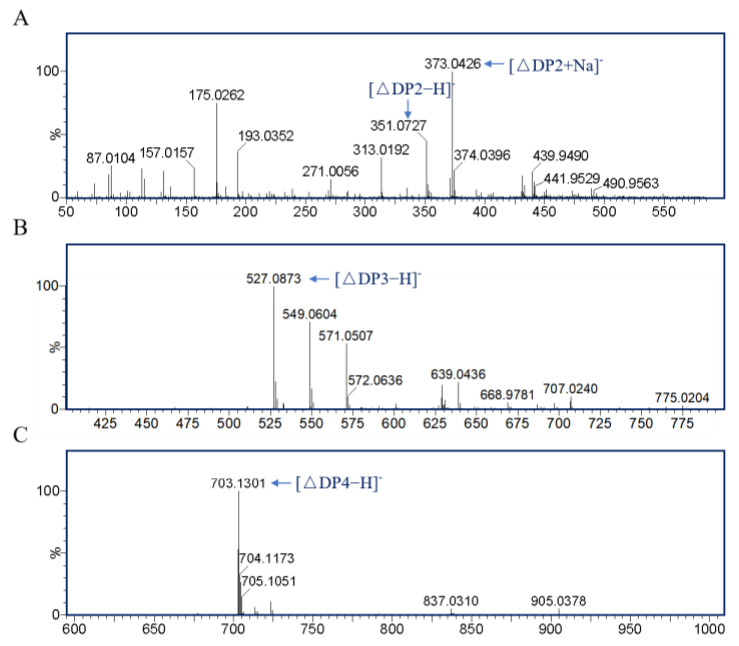
Mass spectrometry (MS) analysis of the peaks in Figure 6. (**A**) MS analysis of the peaks ∆DP2 at 4.6 min in Figure 6B; (**B**) MS analysis of the peaks ∆DP3 at 6.5 min in Figure 6B; (**C**) MS analysis of the peaks ∆DP4 at 7.6 min in Figure 6B.

To analyze the products of PMD, TLC and LC-MS analyses were applied. As shown in Figure 6A, the final products of PMD were determined via TLC analysis. Because of the strict PM substrate specific property of PMD, PM (~99%) was almost completely enzymatically degraded with unsaturated disaccharides, trisaccharides, and tetrasaccharides as main products while the sodium alginate was only partially enzymatically degraded with disaccharides and trisaccharides as main products. Moreover, no degradation product of PG (~99%) could be detected which validated the degradation result of sodium alginate. To further study the products of PMD, LC-MS analysis was applied to monitor the process of enzymatic reaction from the beginning to 24 h. As Figure 6B shows, unsaturated disaccharides (∆DP2) and trisaccharides (∆DP3), which were identified by mass spectrometry (Figure 7A,B), accumulated slowly with the course of the enzymatic reaction, while the unsaturated tetrasaccharides (∆DP4) showed a different trend. ∆DP4 (mass spectrometry identification result shown in Figure 7C) accumulated rapidly at the initial stage of the reaction. Nevertheless, as time was prolonged, they might be further degraded into unsaturated disaccharides. As a result, the peak area of ∆DP4 (24 h) in Figure 6B shows a significant decrease when compared with the peak of 2 h. 

Hence, considering the results of TLC and LC-MS analyses, PMD might be classified into endolytic lyase which eventually yielded unsaturated disaccharides (dominant) and trisaccharides as main products. The result of the final products of PMD (DP 2–3) was consistent with A1-III (PL 5) obtained from *Sphingomonas* sp. A1, which also produced disaccharides and trisaccharides as products [19].

### 3.6. Molecular Docking and Potential Critical Catalytic Residues Identified

**Figure 8:**
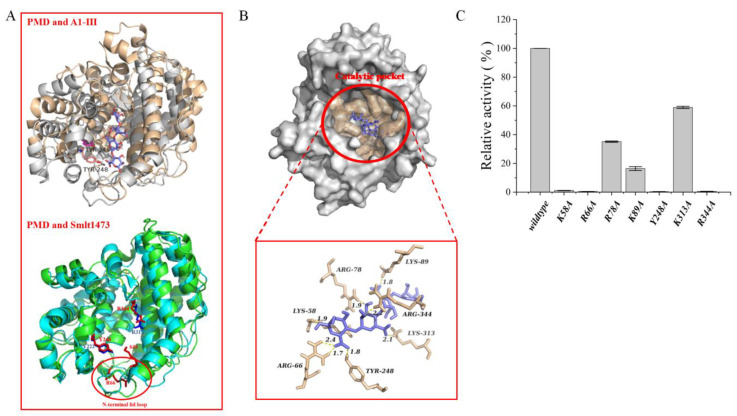
Results of potential critical catalytic residues of the novel alginate lyase (PMD). (**A**) Structure alignment of PMD (gray) and A1-III (wheat, polysaccharide lyase family 5 alginate lyase whose crystal structure has been solved) and the critical catalytic residues of A1-III with aligned residues in PMD (shown as stick; labeled as TYR-246 of A1-III and TYR-248 of PMD); structure alignment of PMD (green) and Smlt1473 (cyan, polysaccharide lyase family 5 alginate lyase whose crystal structure has been solved) and the critical catalytic residues of A1-III with aligned residues in PMD. (shown as stick; labeled as: Y-222 and R312 of Smlt1473, K58, R66, Y-248 and R344 of PMD); (**B**) result of molecular docking of PMD with a tetrasaccharide; (**C**) Results of relative activities of purified mutant enzymes.

As described above, the homology model of PMD was built and the quality of model was evaluated as shown in Figure S4 and S5. To investigate the potential critical catalytic residues of PMD, the software AutoDock (version 4.2.6, San Diego, California, CA, U.S.A) was applied to simulate a model of PMD-tetrasaccharide complex without any artificial residue restrictions except the tetrasaccharide. A total of 1000 complexes were generated finally and ranked by binding energy. The complex with the lowest binding energy was chosen to assist to identify critical catalytic residues. As shown in Figure 8B, a total of six residues located in the catalytic pocket possessing a hydrogen bond with the tetrasaccharide in the complex model were determined to be candidate catalytic residues which were located in a completely conserved region (Figure 2, labeled by red Pentastar). In addition, another candidate residue (Y248) was determined via sequence alignment with A1-III (sourced from *Sphingomonas* sp. A1, PL 5, PDB: 1HV6) whose catalytic residue (Y246) was determined via solving the structure of a complex with trisaccharide [45]. The roles of seven candidate catalytic residues (K58, R66, R78, K89, Y248, K313, and R344) playing during catalytic process were identified with the aid of alanine scanning mutagenesis method via site-directed mutation. The seven mutants were constructed and purified and their relative activities were assayed under the same condition of wildtype. As shown in Figure 8C, mutants K58A, R66A, Y248A, and R344A almost totally lost their activities, while mutants R78A, K89A, and K313A still retained relative activities from about 20% to 60% which insisted residues K58, R66, Y248, and R344 might play a significant role during the catalytic process.

To further analyze the potential roles of these residues, the homology model of PMD was superposed to the structure of A1-III (PL5) and Smlt1473 (PL5), respectively. As shown in Figure 8A, the residue Y248 of PMD was located at the same site as the residue Y246 of A1-III and the residue Y222 of Smlt1473 which suggested that they might possess the same function. Y246 of A1-III was identified as the critical catalysis residues and formed two hydrogen bonds with the substrate [45]. Y222 of Smlt1473 was identified as the catalytic proton donor/acceptor within the active site and formed two H-bonded interactions of 2.9 and 2.7 Ǻ, respectively, with the cleavable glycosidic bond [46]. The residues K58 and R66 were located at the N-terminal lid loop (marked in Figure 8A), in which it was reported that the allosteric effects of sugar binding to it influenced enzyme activity (Smlt1473) [47]. Moreover, it was also reported that the N-terminal loop lid of PanPL (PL5 alginate) played an important role in substrate binding which arched over the active site [26]. Finally, the residue R344 was located at the same site as R312 (in Smlt1473) which was at the surface of the entry site and reported to form a bifurcated interaction of 2.7 and 2.9 Ǻ with the substrate [46].

Therefore, based on the results of site-directed mutation and the literature, it is rationally presumed that the residue Y248 might be the critical catalysis residue and the residues K56, R66, and R344 might play a significant role in the acquisition, translocation, orientation, and positioning of substrates at the catalytic site.

## 4. Conclusions

In this paper, a novel PL 5 family alginate lyase PMD from *Pseudomonas mendocina* ZWU0006 was expressed and identified. PMD only possessed the central *α*/*α* half-barrel catalytic domain. Unlike some PL 5 alginate lyase, the activity of PMD could not be detected without sodium chloride. Meanwhile, PMD performed a great salt-tolerance property, which possessed more than 65% relative activity even under 1 M NaCl. The substrate specificity and product analysis result insisted that PMD might be a strict polyM specificity and endolytic alginate lyase, which might be helpful to produce alginate oligosaccharides with a high M rate. In addition, the potential critical catalytic residues (K58, R66, Y248, and R344) of PMD were also characterized. The detailed study of PMD would supply more information about the properties of the PL 5 family alginate lyase. In summary, the salt-tolerance, strict polyM specificity, pH adaption, and stability properties of PMD made it a potential candidate tool for the directed production of AOS.

## Figures and Tables

**Table 1:** Data of all characterized PL5 family alginate lyases so far.

Name	NCBI Number	Microbial Source	Optimum		Metal Ions Dependence	Substrate Specificity	Product Analysis	Characterization Year	Reference
			Temperature (°C)	pH					
PMD	WP_047587958.1	*Pseudomonas mendocina*	30	7.0	Yes	M-specific	DP2–3	2021	This study
AlgL	AAA71990.1	*Pseudomonas aeruginosa*	-	-	-	-	-	1993	[15]
	AAG06935.1	*Pseudomonas aeruginosa*	-	-	-	-	-	1993	[17]
	AAC04567.1	*Azotobacter vinelandii*	-	8.1–8.4	Yes	M-specific	DP3–4	1998	[18]
algA	BAA33966.1	*Deleya* marina	-	-	-	-	-	1998	[16]
A1-III	BAB03312.1	*Sphingomonas* sp. A1	30	7.2	No	M-specific	DP2–3	1998	[19]
AlgL	CAA11481.1	*Azotobacter chroococcum*	30	7.5	Yes	-	-	1999	[20]
AlgL	AAF32371.1	*Pseudomonas syringae* pv. syringae	42	7.0	No	M-specific	-	2000	[21]
	AAR23929.1	*Pseudomonas* sp. QD03	37	7.5	No	M-specific	DP3–5	2006	[22]
KS-408	AEW23144.1	*Pseudomonas* sp. strain KS-408	20–30	8.0	No	M-specific	DP2–3	2011	[23]
Smlt1473	CAQ45011.1	*Stenotrophomonas maltophillia*	25	9.0	-	M-specific	DP2/4/6/8	2013	[24]
AlgA	AIY22644.1	*Pseudomonas syringae* pv.	30	8.0	No	M-specific	Dp2–5	2014	[25]
PanPL	AJE99968.1	*Pandoraea apista*	-	7.0	-	M-specific	Dp2–3	2022	[26]

^note^ uncharacterized: -.

**Table 2:** Primer sequences used for site-directed mutation.

Variants	Primers Name	Primer Sequences (5′-3′)	T_m_ (°C)
PMDK58A	K58A-F	5′- TACTAGCgctTACGAGGGGTCTAACTCGGCTC-3′	62.5
	K58A-R	5′- CCTCGTAagcGCTAGTAAACTGCAAATCGCCA-3′	60.2
PMDR66A	R66A-F	5′- TAACTCGGCTgctGCAACGCTTAACCGTAAGGC-3′	60.3
	R66A-R	5′- TTGCagcAGCCGAGTTAGACCCCTCGTACTTG-3′	63.4
PMDR78A	R78A-F	5′- GAACTTCgctGAGCAGACAGCCAATATCACACG-3′	62.2
	R78A-R	5′- TCTGCTCagcGAAGTTCTTTTCAGCCTTACGGTT-3′	60.8
PMDK89A	K89A-F	5′- ACACGCCTGGAAgctGAGGCTGGGCGTATGATTACC-3′	61.2
	K89A-R	5′- TCagcTTCCAGGCGTGTGATATTGGCTGTCTG-3′	62.7
PMDY248A	Y248A-F	5′- CCCTTGCTgctCATAATTATGCCCTGCCACCA-3′	60.9
	Y248A-R	5′- ATTATGagcAGCAAGGGCGCGCTGCGAACGAC-3′	63.8
PMDK313A	K313A-F	5′-GGATTACgctTATGCCTGGCTGGCCCCATATT-3′	62.4
	K313A-R	5′-AGGCATAagcGTAATCCTTATGCAGCTCAGCCA-3′	61.4
PMDR344A	R344A-F	5′-ACAGCTTTgctCTTGGGGGCGAAGTTACTCAA-3′	61.8
	R344A-R	5′-CCCAAGagcAAAGCTGTTGAACGGACCGCGCT-3′	61.4

^note^ Mutated codons are underlined.

**Table 3:** Purification of recombinant PMD.

Steps	Total Protein (mg)	Total Volume (mL)	Protein Concentration (mg/mL)	Total Activity (U)	Specific Activity (U/mg)	Yield (%)	Purification (Fold)
Crude enzyme	33.02	15.50	2.13	126.54	3.83	100.00	1.00
Ni-NTA	2.48	2.00	1.24	111.53	44.97	88.14	11.74

**Table 4:** Kinetic parameters of PMD towards alginate and PM.

Substrate	*K*_m_ (mg mL^−1^)	*V*_max_ (μmol s^−1^)	*k*_cat_ (s^−1^)	*k*_cat_/*K*_m_ (mL mg^−1^ s^−1^)
Alginate	4.03 ± 0.68	0.29 ± 0.02	232 ± 16.00	57.57
PM	1.32 ± 0.16	0.28 ± 0.01	224 ± 8.00	169.70

## Data Availability

The date are available from the corresponding author.

## Supplementary Materials

The following supporting information can be downloaded at: https://www.mdpi.com/article/10.3390/foods11213527/s1, Figure S1: SDS-PAGE (12.5%, *w*/*v*) gel showing purified PMD and its mutants; Figure S2: Circular Dichroism (CD) Spectra of PMD; Figure S3: Relative activities of PMD towards PM, sodium alginate and PG; Figure S4: The superposition of the model built and the template; Figure S5: Quality assessment of PMD homology model; Table S1: The percentage of secondary structures calculated from the circular dichroism.
