# Supplementary material for: Characterization of a Novel Polysaccharide Lyase Family 5 Alginate Lyase with PolyM Substrate Specificity
Source: Foods. 2022 Nov 6;11(21):3527. doi: 10.3390/foods11213527 (PMC9655155; doi:10.3390/foods11213527)
Supplement: Supplementary file 1 [file foods-11-03527-s001.zip › foods-1905611-supplementary.pdf]

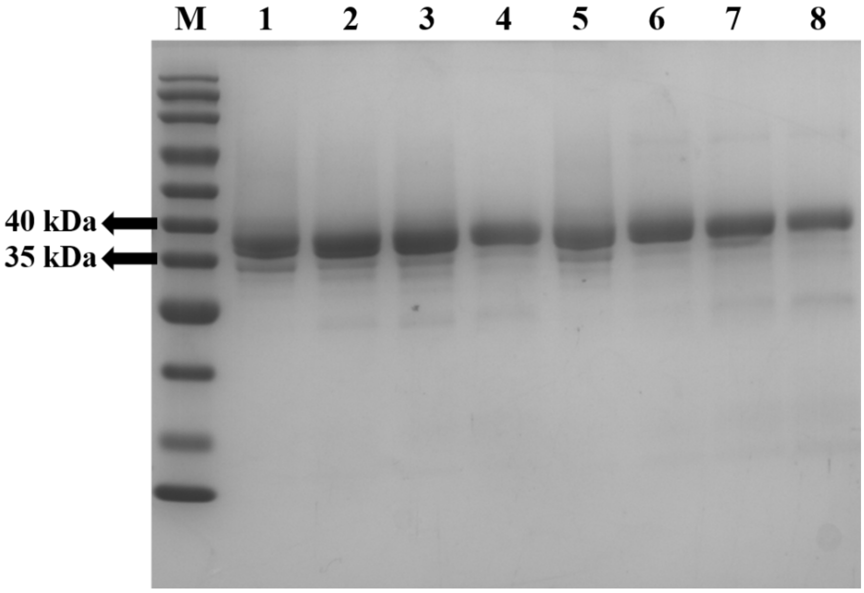

Figure S1. SDS-PAGE (12.5%, w/v) gel showing purified PMD and its mutants. Lane M, molecular weight marker; lane 1, purified PMD; lane 2, purified K58A; lane 3, R66A; lane 4, R78A; lane 5, K89A; lane 6, Y248A; lane 7, K313A; lane 8, R344A.

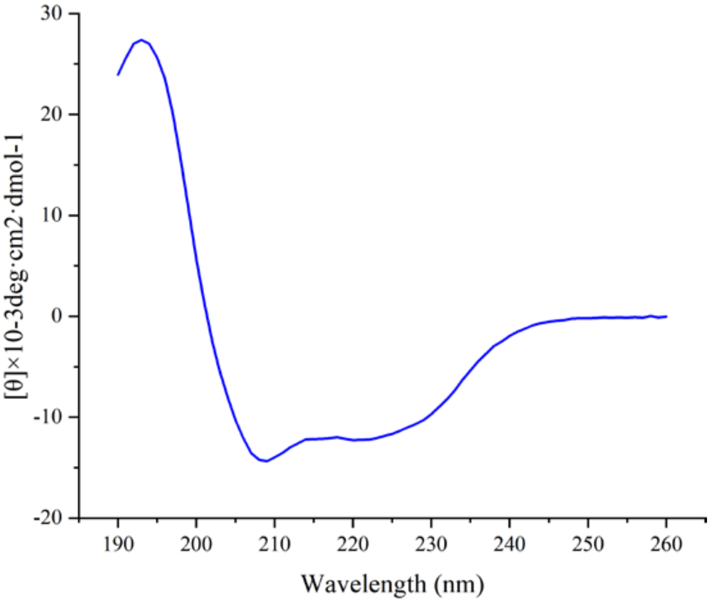

Figure S2. Circular Dichroism (CD) Spectra of PMD.

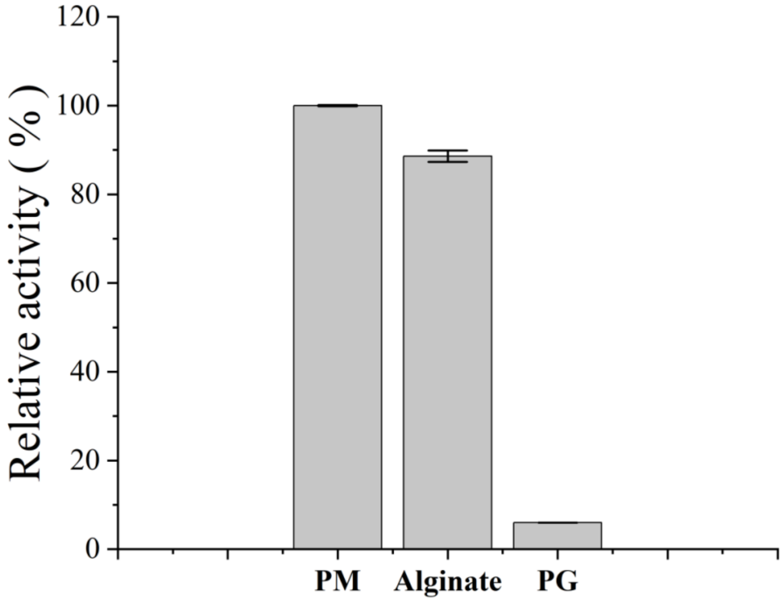

Figure S3. Relative activities of PMD towards PM, sodium alginate and PG (maximum activity was identified as 100%).

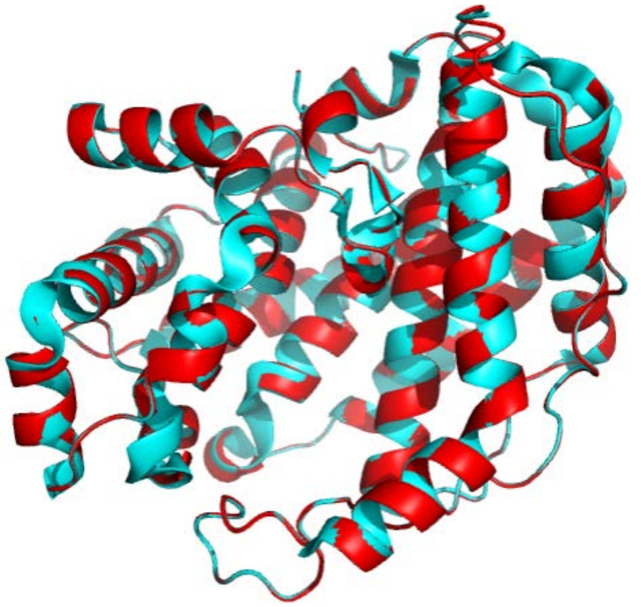

Figure S4. The superposition of the model built and the template. (the model built colored in red and the template colored in cyan)

A B

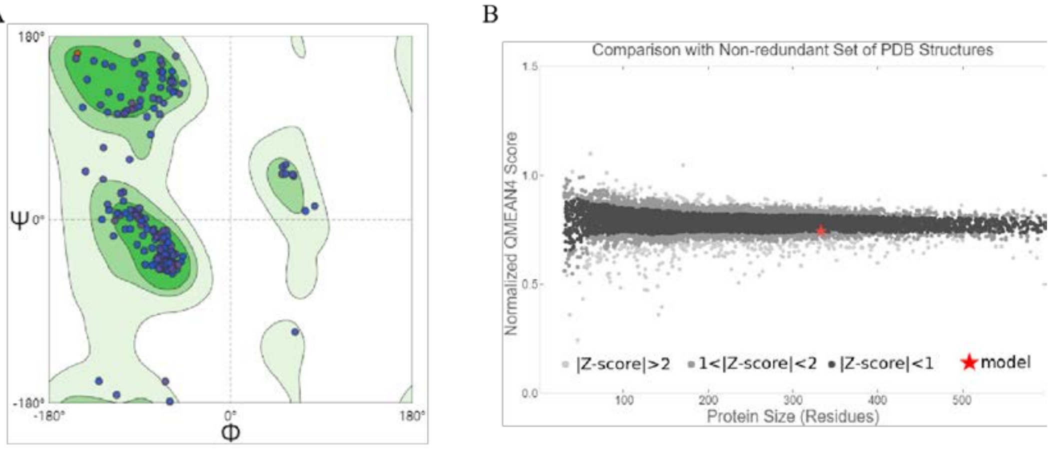

Figure S5. Quality assessment of PMD homology model. (A) Ramachandran Plots of homology model. (B) Quality comparison of homology model

**Composition**	**Helix**	**Antiparallel**	**Parallel**	**Beta-Turn**	**Random**	**Total**
**Proportion (%)**	85.3	0.2	1.7	8.7	4.4	100.3

Table S1. The percentage of secondary structures calculated from the circular dichroism
